# Micro-analytical and molecular approaches for understanding the distribution, biochemistry, and molecular biology of selenium in (hyperaccumulator) plants

**DOI:** 10.1007/s00425-022-04017-8

**Published:** 2022-11-23

**Authors:** Katherine Pinto Irish, Maggie-Anne Harvey, Hugh H. Harris, Mark G. M. Aarts, Cheong Xin Chan, Peter D. Erskine, Antony van der Ent

**Affiliations:** 1 The University of Queensland, Sustainable Minerals Institute, Centre for Mined Land Rehabilitation, Brisbane, QLD 4072 Australia; 2 Department of Chemistry, The University of Adelaide, Adelaide, SA Australia; 3 Laboratory of Genetics, Wageningen University and Research, Wageningen, The Netherlands; 4 The University of Queensland, School of Chemistry and Molecular Biosciences, Australian Centre for Ecogenomics, Brisbane, QLD 4072 Australia

**Keywords:** Genome-scale studies, Selenium metabolism, SMT, Spectroscopy, SULTR, Transcriptomics, X-ray fluorescence elemental mapping

## Abstract

**Main conclusion:**

Micro-analytical techniques to untangle Se distribution and chemical speciation in plants coupled with molecular biology analysis enable the deciphering of metabolic pathways responsible for Se tolerance and accumulation.

**Abstract:**

Selenium (Se) is not essential for plants and is toxic at high concentrations. However, Se hyperaccumulator plants have evolved strategies to both tolerate and accumulate > 1000 µg Se g^−1^ DW in their living above-ground tissues. Given the complexity of the biochemistry of Se, various approaches have been adopted to study Se metabolism in plants. These include X-ray-based techniques for assessing distribution and chemical speciation of Se, and molecular biology techniques to identify genes implicated in Se uptake, transport, and assimilation. This review presents these techniques, synthesises the current state of knowledge on Se metabolism in plants, and highlights future directions for research into Se (hyper)accumulation and tolerance. We conclude that powerful insights may be gained from coupling information on the distribution and chemical speciation of Se to genome-scale studies to identify gene functions and molecular mechanisms that underpin Se tolerance and accumulation in these ecologically and biotechnologically important plants species. The study of Se metabolism is challenging and is a useful testbed for developing novel analytical approaches that are potentially more widely applicable to the study of the regulation of a wide range of metal(loid)s in hyperaccumulator plants.

**Graphical Abstract:**

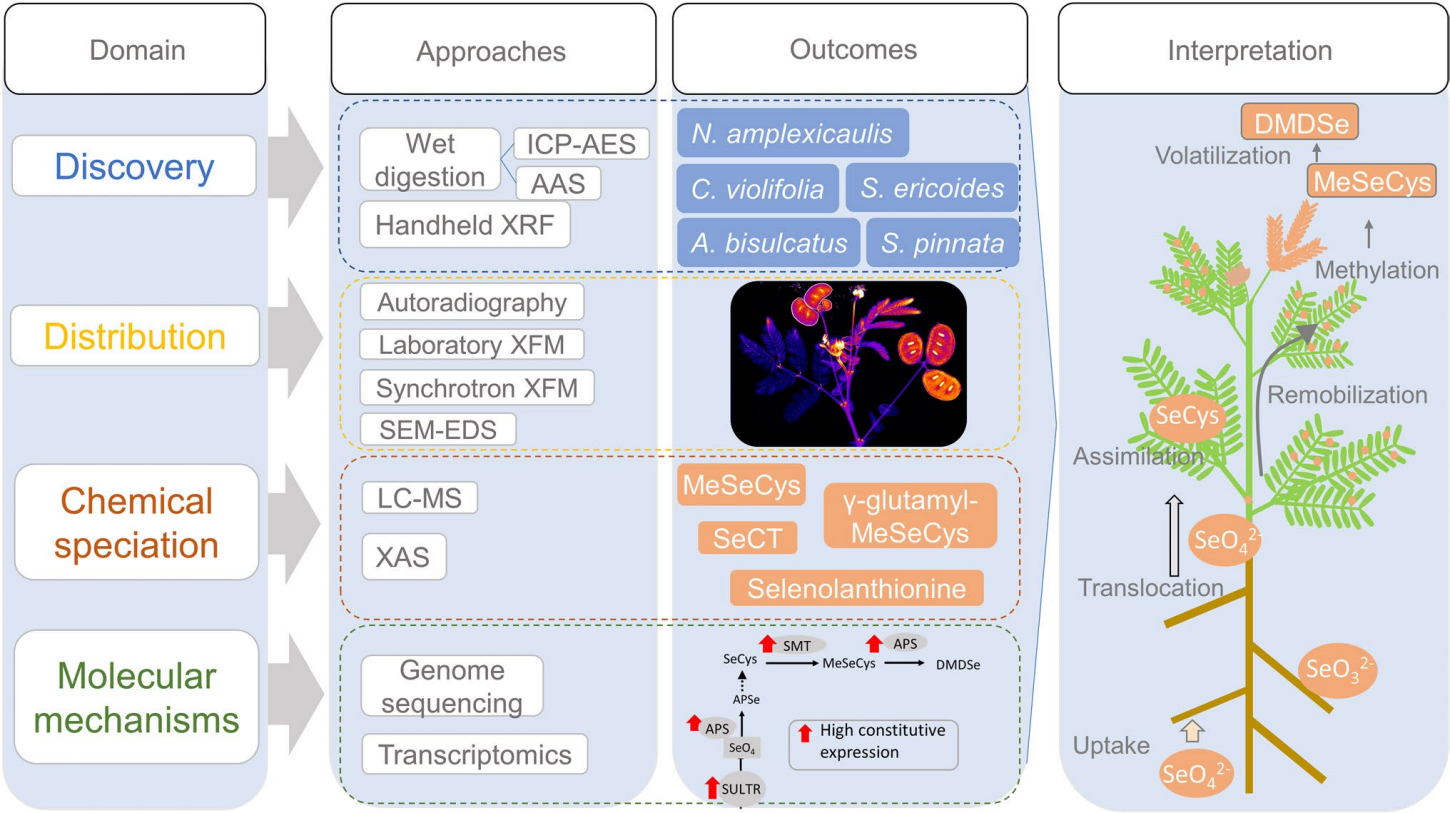

## Introduction

**Fig. 1:**
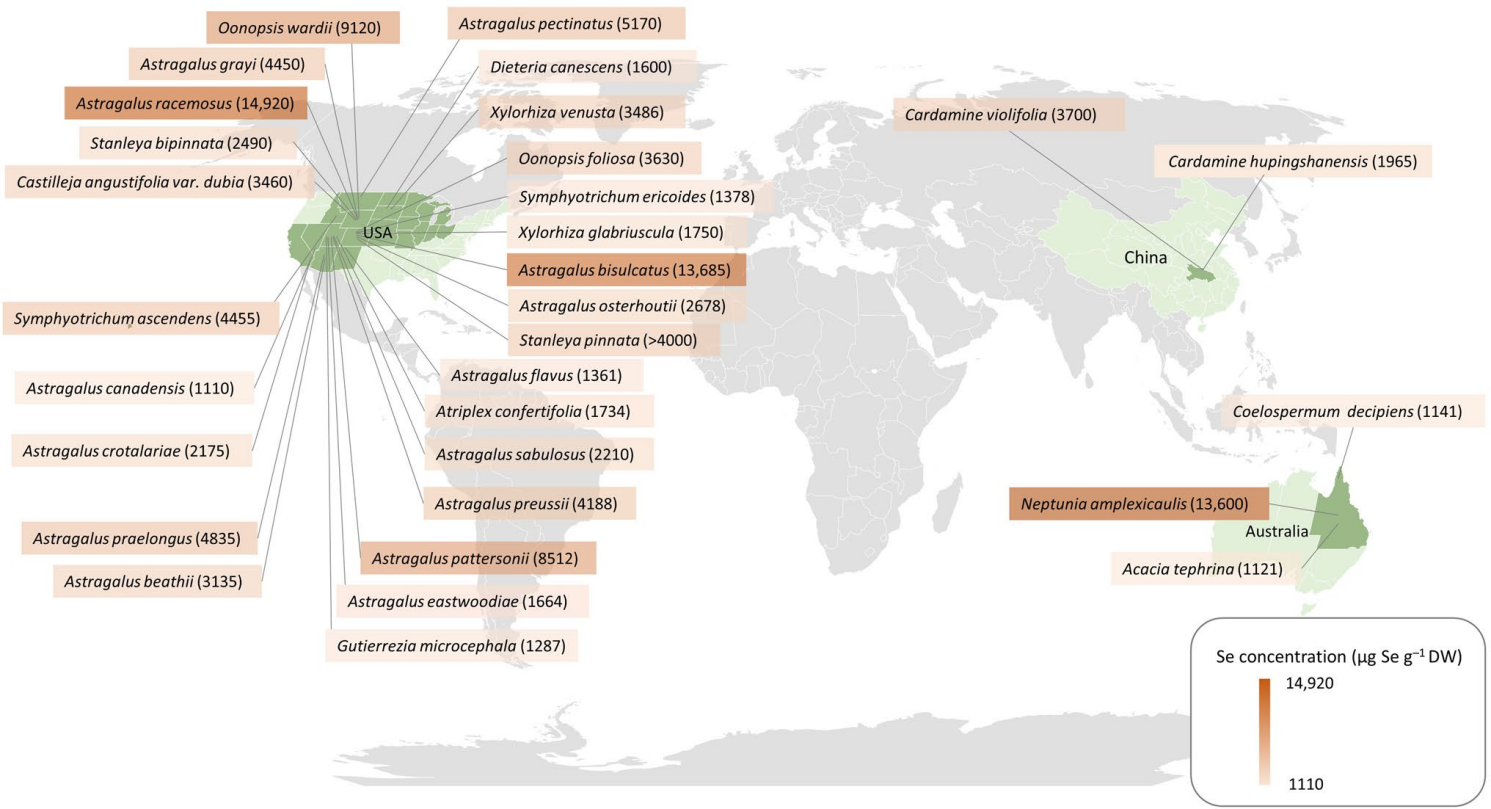
Global selenium hyperaccumulators species (> 1000 µg g^−1^ DW foliar Se) with foliar concentration data from either nature or experimental conditions growing on Se spiked soil (hydroponics data excluded). Concentration and location data from White (2016), Both et al. (2018) and Harvey et al. (2020)

Selenium (Se) is a trace element that is essential for human and animal nutrition (Schwarz and Foltz 1957). It is incorporated in selenoproteins that play important roles in redox balance maintenance, immune response, cognitive health, and the formation of thyroid hormones (Kieliszek and Błażejak 2013; Weekley and Harris 2013). While inadequate intake of Se causes Se deficiency, excess Se can cause a rare condition called *selenosis*, mostly found in livestock from seleniferous areas (Plant et al. 2003; WHO 2009; Winkel et al. 2012; Malagoli et al. 2015; Wu et al. 2015; Rayman et al. 2018). Although Se is not essential for plants, the element can be absorbed and accumulated in plant tissues, which makes them an important source of Se dietary for animals (Dumont et al. 2006). Rare plants species called *hyperaccumulators* can accumulate > 1000 µg Se g^−1^ DW in their shoots whilst non-accumulator plants typically have < 100 µg Se g^−1^ DW (Brown and Shrift 1982; Anderson 1993; Terry et al. 2000) (Fig. 1). Since Se hyperaccumulator plants can attain Se to high concentrations in their living shoot, these plants are potential candidates for phytoextraction; a process in which plants are harvested to remediate a polluted soil and/or for their accumulated trace element, such as Se, and used in the production of dietary supplements (Bañuelos et al. 1997; Haug et al. 2007).

Selenium in the environment is commonly found as selenate (SeO_4_^2−^) and selenite (SeO_3_^2−^) (Sors et al. 2005). Whereas SeO_4_^2−^ is the main Se form in oxic soils, including most cultivated soils (White 2016), SeO_3_^2−^ is prevalent in anaerobic soil environments (Mikkelsen et al. 1989; Fordyce 2013). Plants mainly take up Se as SeO_4_^2−^ through sulphate transporters (SULTRs) (Zhang et al. 2014; White 2016). Once inside the cell, SeO_4_^2−^ can be reduced and incorporated into the amino acids selenocysteine (SeCys) and selenomethionine (SeMet) using the pathway for sulphate reduction and assimilation (Sors et al. 2005; White 2018). The sulphide bridge between two cystine (Cys) residues allows the tertiary structure in proteins, therefore the replacement of Cys for SeCys affects their structure and function (Brown and Shrift 1982). Additionally, at an enzyme active site, the replacement of Cys for SeCys can affect the affinity for the substrate leading to changes in the activity (Van Hoewyk 2013). The first step in the assimilation is the activation of SeO_4_^2−^ to adenosine 5′-phosphoselenate (APSe) by the enzyme adenosine triphosphate sulfurylase (ATPS). APSe is then reduced to SeO_3_^2−^ by adenosine 5′-phosphosulfate reductase (APSR) (Sors et al. 2005). Selenite is reduced to selenide most likely by glutathione or glutaredoxins (Hsieh and Ganther 1975) which is incorporated into SeCys by the Cysteine Synthase complex (CS) (Bogdanova and Hell 1997). Plants can convert SeCys into elemental selenium (Se^0^) by the action of chloroplast-localised cysteine desulfurase (CpNifS) (Pilon-Smits et al. 2002). In a different pathway, SeCys can be converted via SeMet into Se-methyl selenomethionine from where plants can volatilise dimethyl selenide (DMSe) (Lewis and Johnson 1974). In a third pathway described for hyperaccumulators and some non-accumulators, SeCys is methylated by Selenocysteine methyltransferase (SMT) to form methyl-SeCys (MeSeCys), thus avoiding the incorporation into proteins. MeSeCys is then converted into dimethyl diselenide (DMDSe), another volatile compound (Evans et al. 1968). The methylation of SeCys and the synthesis of DMDSe appear as the two main strategies to cope with Se toxicity in Se hyperaccumulators (Pilon-Smits and LeDuc 2009).

Research on the metabolism of Se in plants has been complicated by the complexity of Se biochemistry and the volatile properties of some Se compounds. The development of new techniques has expanded the knowledge on Se metabolism, accumulation, and tolerance in Se hyperaccumulator species. Here, we review the state-of-the-art and current approaches used to discover Se hyperaccumulator plants, to assess the plant distribution and chemical speciation of Se, and to elucidate the molecular mechanisms that underpin Se accumulation and tolerance in plants. These approaches range from microanalytical chemistry to molecular biology covering advanced microscopy using X-ray fluorescence, as well as genome-scale molecular techniques. The study of Se metabolism is not only interesting, but extremely challenging and it, therefore, makes it a useful testbed for developing novel approaches that are applicable to the study of a wide range of metal(loid)s in hyperaccumulator plants. This review concludes with suggested future directions for research to improve the understanding of Se hyperaccumulator plants.

## Discovery of selenium (hyper)accumulation in plants

Plants colonising seleniferous soils have evolved mechanisms of tolerance and strategies to cope with Se toxicity (Cappa et al. 2015). The first Se hyperaccumulator plants were discovered in the 1930s when cattle disease (‘Blind Staggers’ *e.g.* selenosis) was associated with ingesting of high Se concentrations in some plant species in the Western United States (Trelease et al. 1936). These plants occurred on seleniferous soils derived from Cretaceous and Eocene shales (Beath et al. 1934). Of the Se hyperaccumulator plants subsequently discovered in the area, *Astragalus racemosus * has been the species with the highest Se concentrations, attaining up to 14,920  µg Se g^−1^ DW in its leaves (Knight and Beath 1937). Although the genus *Astragalus* (Fabaceae) contains the greatest number (25) of Se hyperaccumulator taxa described to date, Se hyperaccumulation occurs across 45 taxa in six different plant families (Cappa and Pilon-Smits 2014; White 2016), including species from the genera *Stanleya* (Brassicaceae), *Oonopsis*, *Xylorhiza*, *Symphyotrichum* (Asteraceae), *Cardamine* (Brassicaceae), and *Neptunia* (Fabaceae) (Knott and McCray 1959; Rosenfeld and Beath 1964; El Mehdawi et al. 2014; White 2016).

**Fig. 2:**
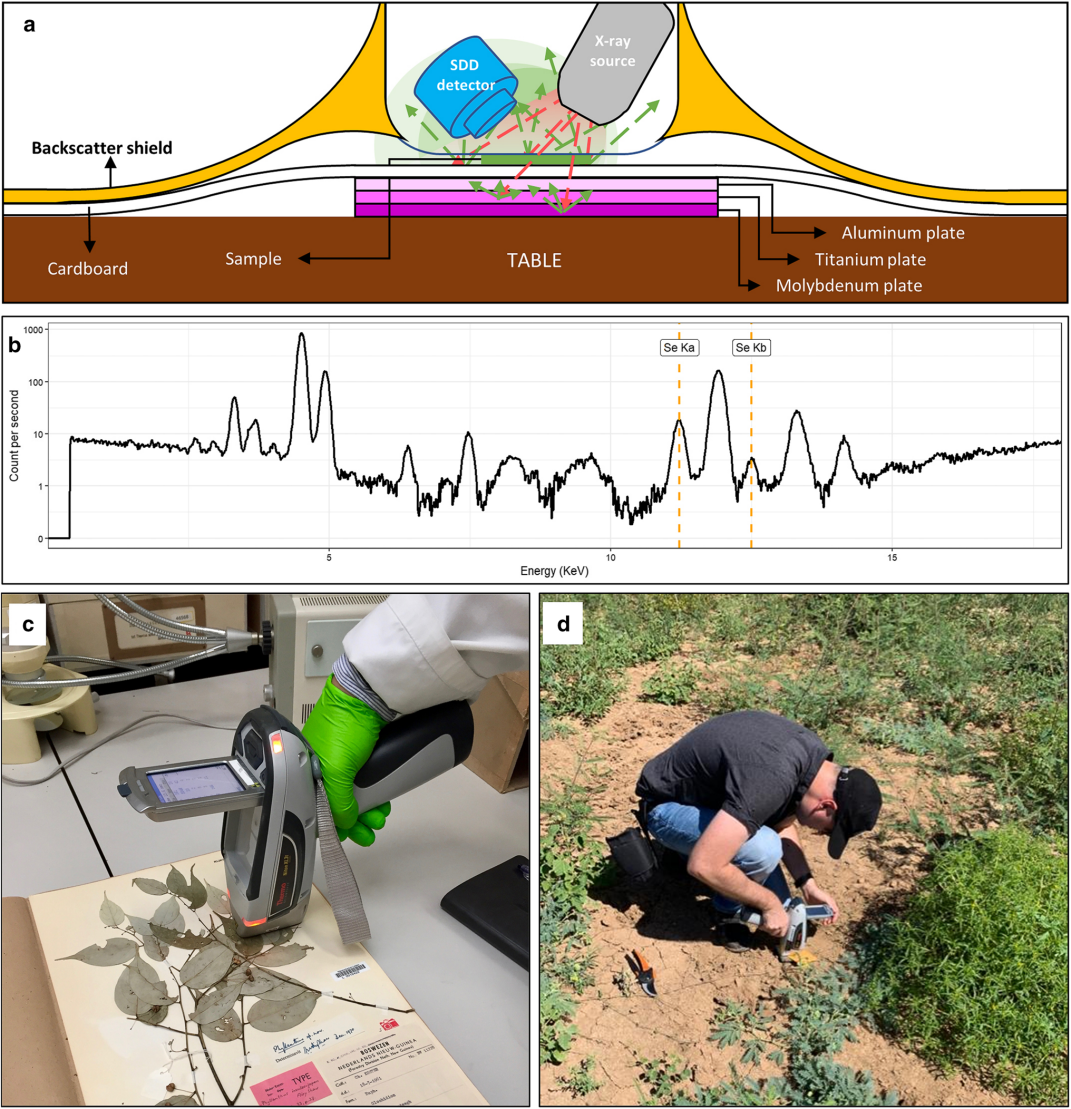
Handheld XRF used in the discovery and analysis of Se hyperaccumulator plants in the field and herbarium. Scheme of X-rays impacting a sample and fluorescent X-rays emitted and recorded by the detector **a**, spectrum of excited fluorescent X-rays to calculate relative Se concentration **b**, example of the use of handheld XRF in herbarium and field samples **c** and **d**, respectively. Schematic panel on top adapted from Purwadi et al. (2021)

The analytical determination of Se concentrations in plant tissues include destructive (*e.g.* ashing or wet acid digestion) and non-destructive (*e.g.* X-ray fluorescence analysis) techniques (Gei et al. 2018; Purwadi et al. 2021). Among the destructive techniques, wet acid digestion, which requires dried and ground plant tissue to be reacted with nitric acid at ~ 125 °C (Shamberger 1983), is still the most commonly used today. After digestion, Se can then be measured in the resulting solution by Inductively coupled plasma atomic emission spectroscopy (ICP-AES) or ICP-mass spectroscopy (ICP-MS) (Reeves et al. 1996, 2007; Fernando et al. 2009; van der Ent and Reeves 2015). In contrast, non-destructive techniques include handheld X-ray fluorescence (XRF) for elemental screening of plant samples (Fig. 2). This technique utilises high-energy X-rays to impact a sample and analyses the spectrum of excited fluorescent X-rays, from which Se and its relative concentration can be determined (Purwadi et al. 2021). The main advantage is that this can be done on herbarium specimen collections, and as such a highly efficient botanical survey can be performed without the high costs, and sometimes complex logistics, of a field expedition (Gei et al. 2018). This approach has already been successful in doubling the number of trace element hyperaccumulator plant species known globally from projects undertaken in New Caledonia, Malaysia, Papua New Guinea and the Neotropics (van der Ent et al. 2019a, b; Do et al. 2020; Gei et al. 2020; Belloeil et al. 2021). Although its potential to find new Se hyperaccumulators has yet to be fully tested, handheld XRF instruments appear as a time- and cost-effective tool for initial discoveries of Se accumulation in plants from existing plant collections held at herbaria (van der Ent et al. 2019a, b).

## Whole plant and tissue/cellular distribution of selenium

The Se metabolism includes the processes of Se accumulation in different forms, storage of Se in different tissues, and eventually (partial) volatilisation (Peterson and Butler 1962; Rosenfeld and Beath 1964; Evans et al. 1968; Lewis and Johnson 1974). The biochemistry of Se affects not only the plant itself but also its ecological partners (El Mehdawi et al. 2011, 2015; Reynolds and Pilon-Smits 2018). Apart from destructive analysis of excised parts of plants, there are a number of in situ and/or in vivo techniques that can be utilised to elucidate the distribution of Se within whole plants and plant organs and cells. Autoradiography was one of the first approaches with high sensitivity that was used to study the ability of plants to uptake and translocate Se to different tissues (Rosenfeld and Beath 1964). In autoradiography, plants are grown in a substrate to which the radioisotope ^75^Se is added and the emitted gamma rays are detected using analogue film or digital detectors (Martin et al. 1971). Although this approach allows the detection of only one element at a time, it facilitates the examination of large samples and hydrated plant tissues, thereby enabling the visualisation of Se distribution in live Se hyperaccumulator plants (Kopittke et al. 2020). Even though this method has been very useful in studying Se hyperaccumulator plants in the past (Rosenfeld and Eppson 1962; Martin et al. 1971; Goodson et al. 2003), regulatory constraints of the use of radioisotopes nowadays have greatly diminished its use. The advent of X-ray based techniques that work by detecting emitted fluorescent X-rays, have gained popularity due to their high-resolution and non-destructive nature and multi-element capability (van der Ent et al. 2018; Kopittke et al. 2018; 2020). The use of synchrotron-based X-ray Fluorescence Microscopy (XFM) has been particularly powerful in the study of Se hyperaccumulator plants (Freeman et al. 2006, 2010). Similar to the handheld XRF, this approach uses X-rays to impact a sample and generates an elemental map with the distribution of the elements of interest. A synchrotron is a very large particle accelerator in which electrons are guided through a storage ring to close the speed of light. At various positions in the storage ring, which can be several km in diameter, brilliant X-rays are produced by insertion devices which are guided to so-called beam stations in which measurements take place. In the beam station, a plant sample is mounted in a plastic holder on a motion stage which moves the sample through the intense micron-sized beam of X-rays with the fluorescent X-rays recorded by a detector. This enables the construction of a pixel array of elemental concentrations *i.e.* ‘elemental maps’. Uniquely, this method offers the ability to measure plant specimens in hydrated (live) state without any sample preparation and has high sensitivity (< 10 µg g^−1^ level) and high spatial resolution (< 1 µm). In essence, a whole live plant or any part of a plant organ or sectioned tissue can be analysed “as is” with XFM. The instrumentation can also determine chemical speciation of selected elements in vivo and even spatially using X-ray Absorption Spectroscopy (XAS), see below for more details (Kopittke et al. 2018). In contrast to analysis of whole plant organs probed at the tissue/cellular-scale, the (cryo)sectioning of samples for subcellular-level analysis is highly challenging due to the risk of significant artefacts, although newer XFM computed tomography (XFM-CT) methods enable to obtain 3D models of elemental distribution in physically intact specimens (van der Ent et al. 2018).

**Fig. 3:**
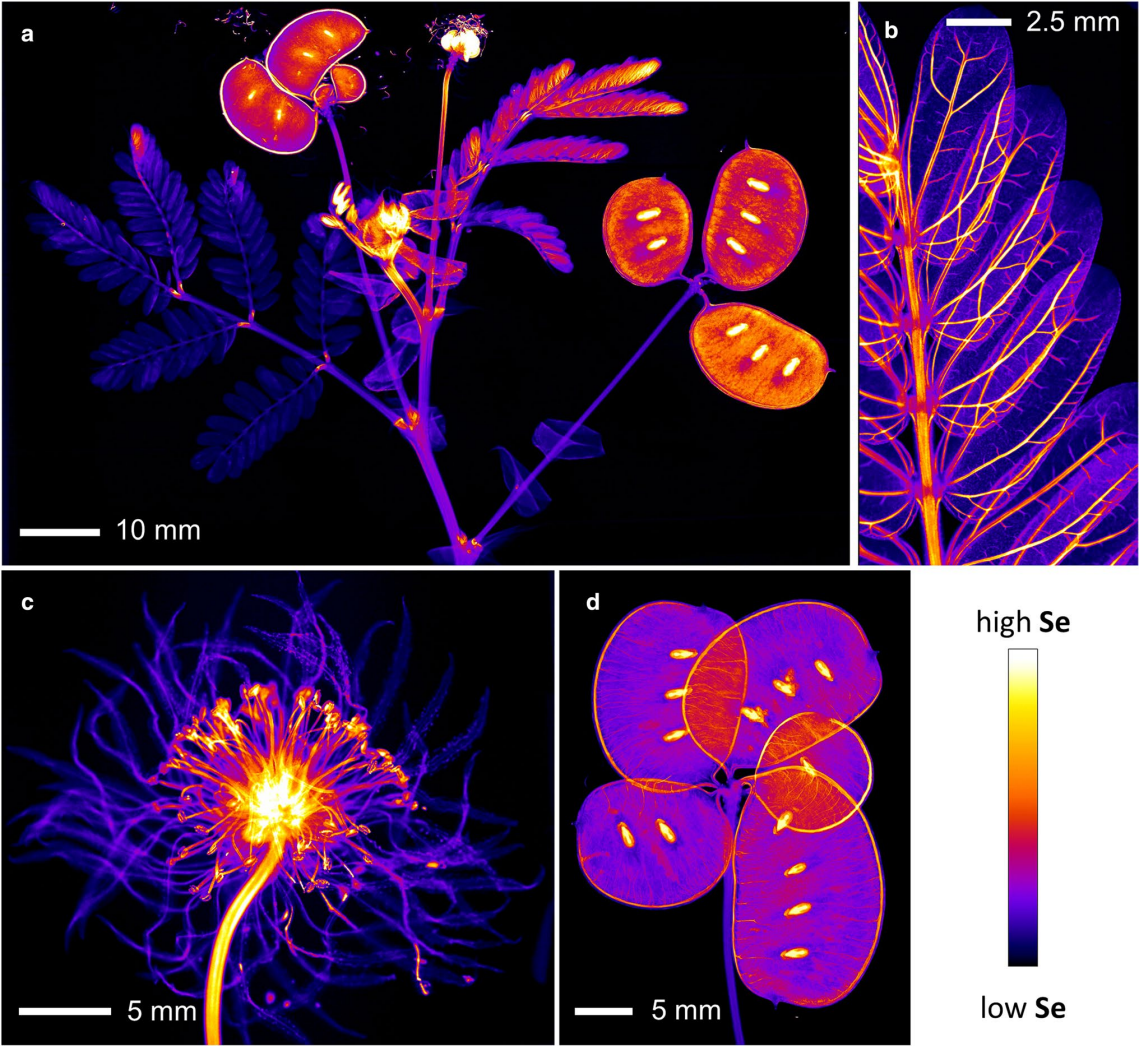
Laboratory-based X-ray Fluorescence Microscopy (XFM) elemental maps of Se in *Neptunia amplexicaulis*. Panels: whole shoot **a**, leaf section **b**, flower **c** and seed pods **d**. Adapted from Harvey et al. (2020)

**Fig. 4:**
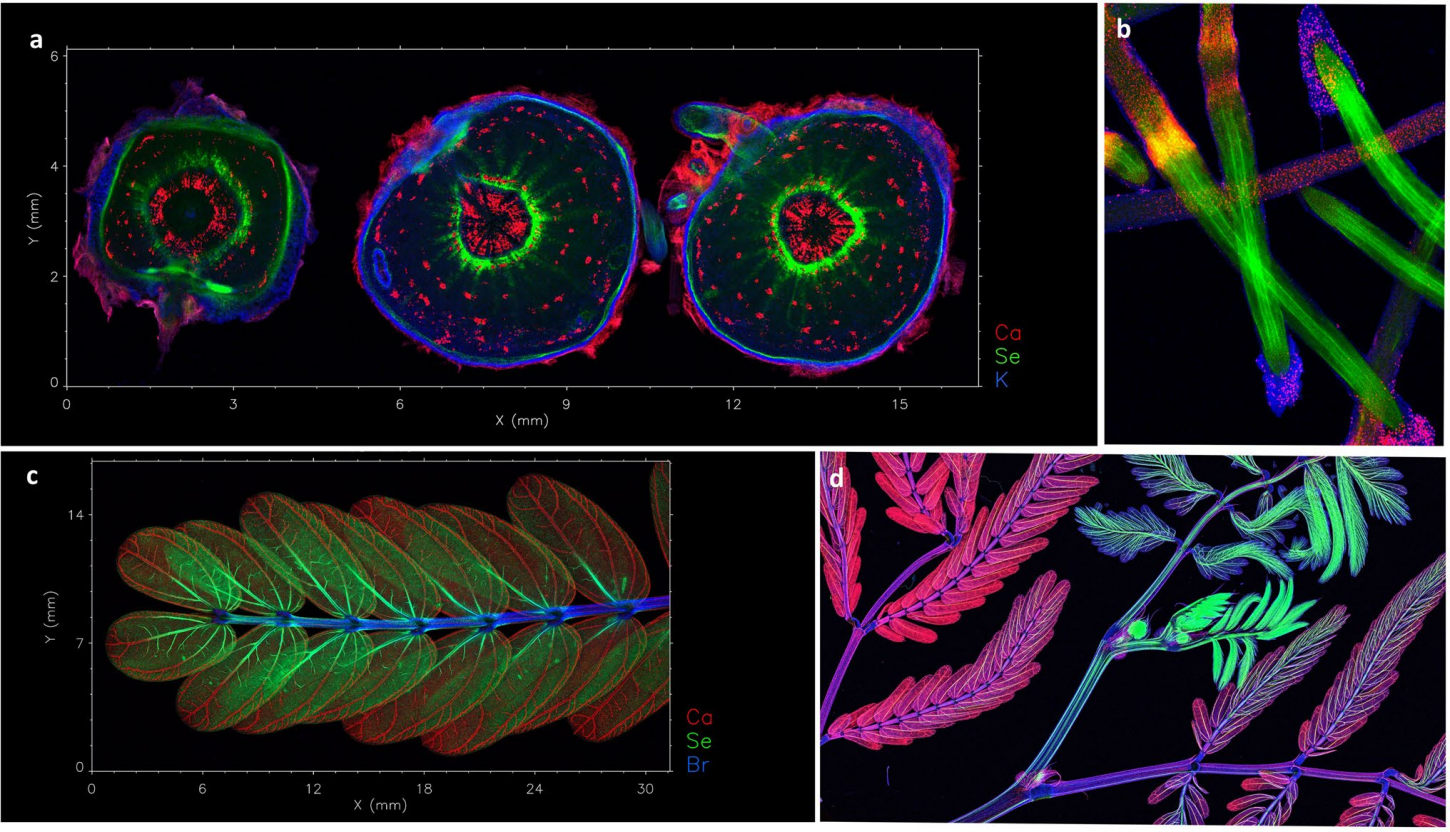
Synchrotron-based X-ray Fluorescence Microscopy (XFM) elemental maps. Panel **a** Ca, Se and K in a fresh/hydrated root cross sections of *Neptunia amplexicaulis;* Panel **b** Ca, Se and Zn in fresh/hydrated root tips of *N. amplexicaulis*; Panel **c** Ca, Se and Br in a fresh leaf of *N. amplexicaulis;* and Panel **d** Ca, Se and K in a fresh/hydrated whole shoot of *Neptunia gracilis.* Previously unpublished data (A. van der Ent) obtained at the X-ray Fluorescence Microscopy beamline of the Australian Synchrotron (part of ANSTO), Australia

More recently, the development of laboratory-based XFM has been demonstrated to be an alternative to synchrotron-based XFM, although it cannot yet compete in terms of spatial resolution or sensitivity and the ability to undertake in situ chemical speciation analysis (Fig. 3) (van der Ent et al. 2018). Recent technological developments have brought capabilities of this method closer to synchrotron-based XFM performance. Similarly, it can analyse specimens in fresh/live state and it offers the ability to scan large specimens (up to 30 × 30 cm) at high resolution (down to 20 µm) with good sensitivity (> 100 µg g^−1^ level) (Fig. 4). The local availability of laboratory-based XFM is particularly attractive for assessing live plants subjected to Se dose treatments (Harvey et al. 2020). Another X-ray based tool that uses the emission of characteristic X-rays to detect Se and other elements is Scanning Electron Microscopy with Energy Dispersive Spectroscopy (SEM–EDS). Although SEM–EDS only allows semi-quantification and has relatively poor limits of detection, it can visualize very small particles of inorganic elements *e.g.* Se in high resolution (1–5 µm) in specific areas of interest, such as on leaf surfaces or in tissue cross-sections (Gei et al. 2018; van der Ent et al. 2018).

The technologies described above have greatly expanded our knowledge about Se distribution in Se hyperaccumulators (see for example, Freeman et al. 2006, 2010; Both et al. 2020; Harvey et al. 2020). Selenium distribution within the plant *e.g.* transport of different Se forms, depends on the plant species, developmental phases, and environmental conditions, such as Se concentrations in soil (Zhao et al. 2005; Li et al. 2008; Renkema et al. 2012). The highest Se concentration has generally been found in reproductive organs such as flowers, fruits, and seeds (Freeman et al. 2006; Quinn et al. 2011; Valdez Barillas et al. 2012; Harvey et al. 2020). This pattern seems to be a characteristic of Se hyperaccumulators because non-accumulators tend to accumulate more Se in the roots (White et al. 2007; Cappa et al. 2014). Another characteristic of Se hyperaccumulators is the high accumulation of Se in young leaves compared to old leaves (Freeman et al. 2006, 2010). Stems and roots have also been identified as Se storage tissues, although the pattern differs among Se hyperaccumulator species. Apart from reproductive organs, the stem is the tissue with the highest Se concentration found in *A. bisulcatus*, followed by young leaves, whereas the roots have the lowest Se concentration (Valdez-Barillas et al. 2012). Additionally, *Neptunia amplexicaulis* accumulates higher Se concentration on average in the seed pods, followed by the young leaves and taproot (Harvey et al. 2020). The distribution pattern in hyperaccumulators, particularly in young leaves and reproductive organs, indicates that Se is transported through the phloem to sink organs during leaf maturation; this pattern has also been associated with defence against herbivore and/or pathogen attacks (Quinn et al. 2010).

When the spatial distribution of Se in leaves was investigated, the Se hyperaccumulators *A. bisulcatus* and *Stanleya pinnata* accumulated Se mostly in the leaf periphery. However, while in young leaves of *A. bisulcatus* Se was predominantly found in their trichomes, in young leaves of *S. pinnata* Se was localised in the epidermal cells near the leaf edges, in structures similar to vacuoles (Freeman et al. 2010). As observed in *S. pinnata*, low Se accumulation in trichomes has also been found in *N. amplexicaulis*, although with high Se concentration in the vascular bundles (phloem) rather than in the leaf lamina (Harvey et al. 2020). The distribution of Se in the leaf edge of Se hyperaccumulators suggests a specific Se sequestration into these tissues, since this pattern has not been observed for other elements (Freeman et al. 2006). Furthermore, the localization of Se in the leaf periphery may be a distinguishing characteristic for Se hyperaccumulators, as this pattern is not observed in the non-accumulators *B. juncea* and *Arabidopsis thaliana*, for which Se is distributed in the vascular tissues and mesophyll cells (Van Hoewyk et al. 2005).

## Unravelling chemical speciation of selenium in plants

Establishing the distribution of various Se chemical forms within a plant is a powerful method to uncover the pathways implicated in Se metabolism and the molecular mechanisms that underpin tolerance to Se toxicity (Freeman et al. 2006, 2010; Valdez-Barillas et al. 2012; Both et al. 2020). The chemical speciation of Se in plants can be analysed using synchrotron-based X-ray Absorption Spectroscopy (XAS), a non-destructive method that utilises a synchrotron X-ray beam to impact a sample which generates an X-ray absorption spectrum specific to the target element and the binding energies of its electrons (Abraham et al. 2020). The XAS spectrum can be divided into two main regions: the X-ray Absorption Near Edge Structure (XANES) and the Extended X-ray Absorption Fine Structure (EXAFS) energy regions. The XANES energy region of the generated spectrum indicates the coordination environment of the Se atom, and therefore can discriminate among Se species by revealing different spectra characteristic for the chemical forms of Se (Weekley et al. 2013; Weekley and Harris 2013). However, not all species can be identified by XANES, as several organic amino acids of Se (including SeMet, SeCys and MeSeCys) possess the same C-Se-C compound structure and generate essentially identical spectra; in most cases XANES is useful in distinguishing between inorganic and organic compounds (Weekley et al. 2013). Details of the distance, number and type of atoms around the central absorbing atom can be obtained through EXAFS analysis and has been able to identify the chemical state of Se species such as Se^(0)^, Se-Se and Se-S compounds (Wiramanaden et al. 2010; Weekley et al. 2014; Abraham et al. 2020). Although XAS analysis provides insight into the chemical speciation between organic and inorganic forms of Se, the most popular tool to investigate Se speciation is Liquid Chromatography coupled to Mass Spectrometry (LC–MS), an analytical technique based on the separation of target compound based on their size, followed by mass-spectrometry to separate co-elutants according to mass-to-charge (m/z) ratio (Katajamaa and Orešič 2005; Xiao et al. 2012). Although LC–MS has allowed the identification of the different organic forms of Se, methods based on chromatography require extensive sample preparation that may affect speciation for which Se compounds are notoriously sensitive (Weekley et al. 2013).

The pathway of Se within hyperaccumulators ends with the volatilisation of DMSe and DMDSe, as part of the mechanism to remove Se and prevent toxicity (Draize et al. 1935; Lewis et al. 1966; Evans et al. 1968; Terry et al. 1992; Zayed et al. 1998; Terry et al. 2000; Van Huysen et al. 2003). To measure the rate of DMSe and DMDSe production, volatile compounds are usually collected in a trap (*e.g.* a bottle containing an alkaline solution) from a growth chamber where a plant is grown in Se-dosed nutrient solution. Selenium content is analysed using Atomic Absorption Spectroscopy (AAS), High Performance Liquid Chromatography (HPLC) coupled to Gas Chromatography (GC), or GC–MS, and, if ^75^Se isotope is applied to the nutrient solution, scintillation detectors can be used to measure radioactivity and hence Se concentration.

Chemical speciation mapping of Se has been undertaken in *A. bisulcatus* and *S. pinnata* (Pickering et al. 2000; Freeman et al. 2006; Valdez-Barillas et al. 2012), and more recently in *N. amplexicaulis* (Harvey, unpublished) and *Cardamine violifolia* (Both et al. 2020). These studies have shown that the Se chemical speciation varies among organs and depends on the habitat where the plant was grown due to the influence of microbial interactions on the forms of Se within the plant (Valdez-Barillas et al. 2012). The main forms of Se are predominantly organo-seleno compounds, such as MeSeCys; *γ*-glutamyl-MeSeCys (*A. bisulcatus*) or selenocystathionine (SeCT; *S. pinnata*) in young leaves (Freeman et al. 2006, 2010). LC–MS analyses of *A. bisulcatus* tissues collected from its natural seleniferous habitat revealed that 50% of the Se contained in the stem was organic Se (C-Se-C), with the remainder elemental Se and SeO_3_^2−^, whilst flowers accumulated primarily C-Se-C (90%) with small fractions of Se^(0)^ and SeO_3_^2−^(Valdez-Barillas et al. 2012). A high proportion of organic Se in hyperaccumulator plants suggests an active sulphate/selenite assimilation pathway to convert SeO_3_^2−^ into organo-seleno compounds (Schiavon and Pilon-Smits 2017). Further Se chemical speciation studies on *A. bisulcatus* and *S. pinnata* growing in its natural habitat showed that up to 35% of Se was in the form of elemental Se (Se^0^) which has been attributed to microbial interactions with Se-reducing bacteria (Lindblom et al. 2013). XANES analyses on young *N. amplexicaulis* revealed that MeSeCys and SeMet occur in similar proportions in young leaves, while selenodiglutathione (Se(GSH)_2_) was the main Se chemical species in the root (40%) (Harvey et al. 2020). Studies performed on the Se hyperaccumulator *C. violifolia* and the non-accumulator *Cardamine pratensis* revealed that the main chemical form of Se in the hyperaccumulator were C-Se-C forms, whilst the non-accumulator contained more SeO_4_^2−^ (Both et al. 2020). Similar observations have been reported when comparing *A. bisulcatus* and *S. pinnata* to related non-accumulator species, with the non-accumulator plants containing a higher proportion of inorganic of Se (Freeman et al. 2006; Alford et al. 2014).

In Se hyperaccumulator plants, the observed organic forms of Se in their young leaves and the inorganic forms (SeO_4_^2−^) in old leaves suggest a translocation from old leaves via the phloem in the form of MeSeCys (Freeman et al. 2006). Further identification of Se chemical species have been performed in leaves of Se hyperaccumulators and XANES and LC–MS analysis of *A. bisulcatus* trichomes revealed a high proportion of Se in organic form, specifically MeSeCys and *γ*-glutamyl-MeSeCys (Freeman et al. 2006). This suggests that trichomes in this species were a site for sequestration and storage, or for the synthesis of organo-seleno compounds (Freeman et al. 2006). Apart from trichomes, the presence of SeO_4_^2−^, SeO_3_^2−^, MeSeCys, and γ-glutamyl-MeSeCys is suggestive of SeO_4_^2−^ reduction and for incorporation of Se into organo-seleno compounds in young leaves (Freeman et al. 2006). Around the edges of *S. pinnata* young leaves, Se was present as MeSeCys (88%) and SeCT (12%) (Freeman et al. 2006). Selenium chemical speciation has also been investigated in seeds of *A. bisulcatus* and *S. pinnata*, where XANES analysis has identified SeCys in *S. pinnata*, and MeSeCys and γ-glutamyl-MeSeCys in *A. bisulcatus* (Freeman et al. 2012)*.*

Volatilisation experiments to measure the rate of DMDSe or DMSe production have shown that hyperaccumulator plants are able to eliminate Se through expelling it in gaseous form through the leaves (Zayed et al. 1998; de Souza et al. 2000; Terry et al. 1992, 2000;). The first findings of Se volatilization were reported in *A. bisulcatus* (Draize et al. 1935). Further studies on the Se hyperaccumulator *Astragalus racemosus* and the non-accumulator *Medicago sativa* revealed that the synthesis of volatile Se compounds is not restricted to Se hyperaccumulator species alone (Lewis et al. 1966). In this case, both species were able to produce DMDSe or DMSe even at low Se doses, and the increased rates of volatilisation are correlated to Se concentrations in plant tissues and the chemical form of Se that was applied (Lewis et al. 1966; Terry et al. 1992; Zayed et al. 1998). Additional studies have shown that Se volatilisation occurs in the leaves mainly as DMSe in crop species (Lewis 1974; de Souza et al. 2000) and as DMDSe in Se hyperaccumulator plants (Evans et al. 1968). Studies aimed to decipher the metabolic pathways of Se volatilisation revealed an increased volatilisation rate in transgenic *B. juncea* that overexpressed cystathionine-γ-synthase (CGS) (Van Huysen et al. 2003).

## Genome-scale approaches for studying selenium metabolism

The molecular biology underlying Se acquisition, transport and metabolism can be investigated using genome-scale approaches (Corso et al. 2020). However, genome-scale data remain scarcely available from Se hyperaccumulator plants (see Huang et al. 2021). Given the diversity of the taxa involved, generation of genome data requires de novo assembly and ab initio gene prediction. The continuing advancement and increased affordability of high-throughput sequencing technologies is enabling data generation at lower costs and at scale previously unimaginable. Short-read sequencing data (*e.g.* from Illumina) that are more cost-efficient can be combined with long-read sequencing data (*e.g.* from PacBio and Oxford Nanopore) that are better in resolving repetitive sequence regions in a hybrid approach to generate a more-contiguous assembly (Zimin et al. 2017; Antipov et al. 2016). Especially if such assembled whole-genome sequence is annotated with the location of (predicted) genes, based on whole transcriptome sequences, it can be a very valuable genetic tool for comparative genome analysis of Se hyperaccumulators and closely related non-accumulators. Comparison of whole-genome sequences elucidates conservation and loss of (micro)synteny, providing clues on genome evolution underlying phenotypic differences between species (Hammond et al. 2006; Weber et al. 2006). These data also provide valuable information on small sequence variations *e.g.* single nucleotide polymorphism, and insertion-deletion, to be used as molecular markers, as well as larger structural variations *e.g.* gene copy-number variations, and large chromosomal rearrangements contributing to phylogenetic divergence and potential acquisition of new traits (Talke et al. 2006; van de Mortel et al. 2006). Full-length transcripts, to be used in genome annotation and comparative transcriptomics, can be achieved using long-read sequencing technology, *e.g.* through the reconstruction of transcript isoforms (Gao et al. 2019). Transcriptome, proteome and/or metabolome profiles from tissues grown in distinct conditions or treatments enable comprehensive assessments of differentially expressed biomolecules (*i.e.* genes, proteins, or metabolites) and their functions in metabolic pathways.

The use of genome-scale approaches is enhancing our understanding of the molecular responses and mechanisms that underpin Se metabolism in the hyperaccumulators. In *Cardamine enshiensis*, the analyses of genome, transcriptome and metabolome data revealed a whole-genome duplication event and other segmental duplications, an enrichment of functions implicated in Se metabolism pathways among the duplicated genes, and changes in the patterns of chromatin interactions in response to Se treatment (Huang et al. 2021). In an early transcriptome analysis (Freeman et al. 2010), genes coding for distinct *SULTRs* that are implicated in uptake and transport of sulphate and SeO_4_^2−^, were found to be constitutively highly expressed in roots and shoots of the Se hyperaccumulator *S. pinnata*, in contrast to the secondary accumulator *Stanleya albescens*. This is considered an advantage for Se and S uptake and has been related to an increase in Se or S concentration in the plant (Cabannes et al. 2011; El Mehdawi et al. 2018; Wang et al. 2018). Furthermore, hyperaccumulators and non-accumulators from the genus *Astragalus* have been shown to have a high constitutive expression of *SULTRs*, regardless of the Se or S concentration in the substrate (Cabannes et al. 2011). The concentrations of Se and S in the substrate do, however, often have an impact on the regulation of *SULTRs*, indicating that high constitutive expression is not the rule for all hyperaccumulator plants, and not for all *SULTRs* (White et al. 2004; Cabannes et al. 2011; Schiavon et al. 2015; El Mehdawi et al. 2018). For example, studies of *S. pinnata* exposed to 20 µM of SeO_4_^2−^ showed a reduction in the expression of genes encoding *SULTR1.1* and *SULTR1.2* responsible for the uptake of SeO_4_^2−^, compared to control plants grown without Se (Schiavon et al. 2015). A more-recent study of the hyperaccumulator *C. violifolia* revealed that eight *SULTR* genes are upregulated in high Se conditions compared to the control conditions, suggesting that these genes might contribute to elevated Se uptake and translocation in this species (Rao et al. 2020).

**Fig. 5:**
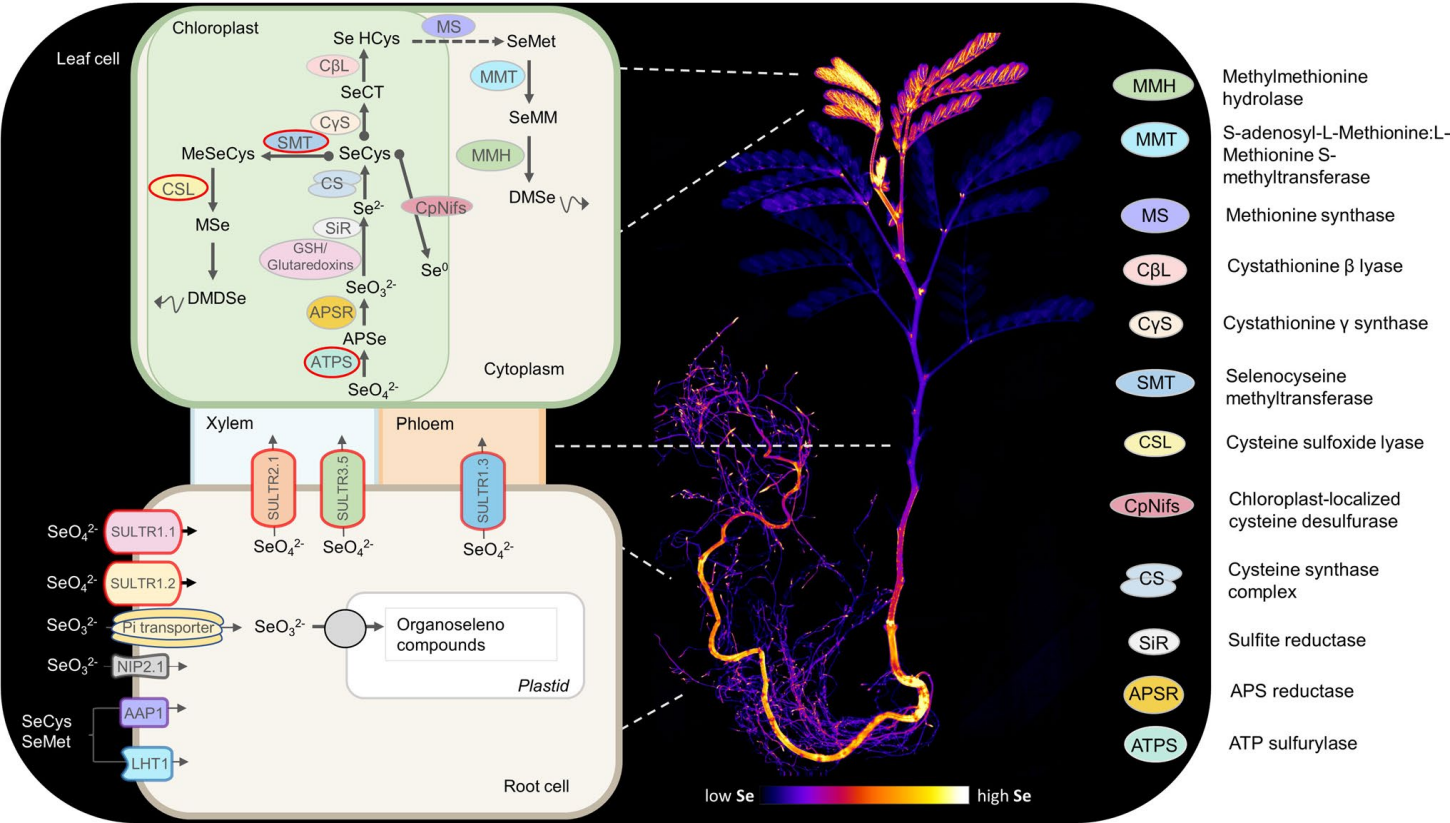
Schematic model of selenium uptake, transport, and metabolism in Se hyperaccumulator plants and map of Se distribution in *Neptunia amplexicaulis* obtained with laboratory-based X-ray fluorescence microscopy (Harvey et al. 2020). Red circles indicate high constitutive gene expression. *SULTR* sulfate/selenate transporter; *Pi transporter* phosphate inorganic transporter; *NIP2.1* Aquaporin channel 2.1; *AAP1* amino acid permease; *LHT1* *AAP1* homolog; *APSe* denosine phosphoselenate; *GSH* glutathione; *SeCT* Selenocystathionine; *Se HCys* Selenohomocysteine; *SeCys* Selenocysteine; *SeMet* Selenomethionine; *MSe* Methaneselenol; *SMM* Se-methyl selenomethionine; *DMSe* Dimethylselenide; *DMDSe* Dimethydiselenide

Once Se is transported into the cell, it can be assimilated into amino acids using S assimilation pathways (Fig. 5) (Schiavon et al. 2017; White et al. 2004; Sors et al. 2005; Bulteau and Chavatte 2015). Transcriptome studies have shown a high constitutive expression of these pathways in Se hyperaccumulators, specifically the enzyme ATPS that catalyses the reduction of SeO_4_^2−^ to form APSe (Leustek 1994; Sors et al. 2005; Pilon-Smits et al. 2009; Schiavon et al. 2015). The gene coding for the isoform ATPS2 that is localised in both the plastid and cytosol showed a higher expression in *S. pinnata* roots and leaves compared to non-accumulator *Stanleya elata*, implicating a molecular response related to hypertolerance and hyperaccumulation in *S. pinnata* (Schiavon et al. 2015; Wang et al. 2018). Selenocysteine methyltransferase (SMT) that methylates SeCys to form MeSeCys has been found to be constitutively highly expressed in hyperaccumulators (Pickering et al. 2003; Pilon-Smits 2012). Although SMT has been identified in several *Astragalus* species including non-accumulators, its functional isoform has been found only in Se hyperaccumulator plants (Neuhierl and Bӧck 1996; Neuhierl et al. 1999; Sors et al. 2009), explaining the high proportion of MeSeCys in these species (Neuhierl et al. 1999; Pickering et al. 2003; Sors et al. 2005; Freeman et al. 2006, 2010). Furthermore, the upregulation of antioxidant activity is thought to be a strategy to cope with oxidative damage caused by Se excess (Freeman et al. 2010). Moreover, the constitutive up-regulation of genes associated with the biosynthesis of or response to phytohormones, *e.g.* methyl jasmonic acid, jasmonic acid, ethylene, and salicylic acid (SA) in *S. pinnata* shoots and roots suggests that the signalling of defence-related phytohormones is tightly linked to Se metabolism (Freeman et al. 2010).

A recent transcriptome analysis of the Se hyperaccumulator *C. violifolia* revealed different metabolic pathways implicated in the Se metabolism and detoxification in this species (Rao et al. 2020). The involvement of adenylyl-sulphate kinases and a phosphoadenosine phosphosulfate reductase family protein was postulated as an alternative pathway to reduce SeO_4_^2−^ to SeO_3_^2−^ in *C. violifolia*, due to their up-regulation in the plants grown in Se-enriched medium (Rao et al. 2020). Furthermore, homocysteine S-methyltransferase (HMT) that participates in the pathway to convert homocysteine into methionine in plants was suggested as an analogue of SMT due to their shared sequence similarity, thus HMT may also be involved in the methylation of SeCys (Lyi et al. 2007). The degradation of SeMet catalysed by Met-γ-lyase (MGL), and the degradation of SeCys into elemental Se mediated by Cys desulfurase, are likely pathways that underpin mechanisms of tolerance in *C. violifolia* (Rao et al. 2020).

Importantly, genome-scale techniques provide the basis for functional validation of candidate genes, *e.g*. via transgenic approaches to reconstruct a working model of Se metabolism in plants (Pilon-Smits et al. 1999; Sors et al. 2005; LeDuc et al. 2006; Van Hoewyk 2013). The overexpression of the APSR enzyme in *A. thaliana* increased Se flux through the plant and SeO_4_^2−^ reduction into organic forms (Sors et al. 2005); the overexpression of CpNifS increased tolerance and accumulation of Se (Van Hoewyk 2013). Several genes coding for enzymes implicated in Se tolerance have been described in *Brassica juncea*, and the overexpression of *ATPS1* in *A. thaliana* were shown to enhance the reduction rate of SeO_4_^2−^ into organic forms (Pilon-Smits et al. 1999); increased accumulation of Se was observed when *APSR* and *SMT* were simultaneously overexpressed (LeDuc et al. 2006). Additionally, the constitutive expression of cystathionine-γ-synthase (CγS) from *A. thaliana* in *B. juncea*, was correlated to an increase in the Se volatilisation rate (Van Huysen et al. 2003). These studies support the role of APSR, ATPS, CpNifS and SMT in the mechanisms of tolerance and accumulation in hyperaccumulators, and in genetically engineered non-accumulators.

## Conclusions and outlook

Current research using microanalytical and molecular biology techniques are enhancing our understanding of Se accumulation and tolerance in hyperaccumulator plants (Table 1). These plants exhibit enhanced Se uptake and translocation rates from the root to the shoot compared the non-accumulators, patterns associated with a constitutively upregulation of genes coding for sulphate transporters. Chemical speciation studies have revealed that C-Se-C compounds, such as MeSeCys, are the prevalent form of Se in hyperaccumulators, compared to inorganic Se forms in non-accumulator plants. This is supported by the consecutively high expression of genes encoding for ATPS and SMT in hyperaccumulators plants which implicate an effective strategy to cope with Se toxicity. Studies on the distribution of Se in hyperaccumulator plants revealed Se sequestration in leaf margins, vacuoles and trichomes, a pattern distinct from that of other elements including S. Further research is needed to determine whether these strategies involve specific transporters and pathways for Se. The knowledgebase gained from this body of work provides the basis for future research to enhance our understanding of the biology, biochemistry, ecophysiology, and evolution of Se hyperaccumulator plants. Insights from the patterns of Se distribution and chemical speciation coupled to genome-scale data from a more diverse range of hyperaccumulator plants will further enhance the model of molecular and evolutionary mechanisms that underpin Se metabolism and tolerance in plants. The integration of microanalytical and genome-scale molecular biology techniques provides a powerful platform to discover novel gene functions or metabolic pathways, as well as conserved (and unique) genome and gene features, in distinct lineages of hyperaccumulator plants. For example, comparative studies of genome and transcriptome coupled with analysis of the chemical speciation in specific tissues will reveal the location where the key steps of Se metabolism are taking place in the plant. In addition, the largely unexplored role of root-associated microbiomes in Se accumulation, particularly in legume hyperaccumulators (such as *Astragalus* ssp. and *N. amplexicaulis*), can be further investigated using metagenomic approaches, in which microbial diversity and their involvement in Se acquisition in the plant can be assessed based on metagenome-assembled genomes and/or meta-transcriptome data. The advent of rapid non-destructive methods will enable the discovery of novel Se hyperaccumulator taxa via high-throughput of herbarium collections, and how Se hyperaccumulation impacts evolution and diversification of these taxa. Although competitive access to synchrotron facilities presents a limitation for many researchers, the development of cutting-edge laboratory-based instrumentation for elemental mapping will partly meet this demand. Overall, the analytical and molecular approaches presented here will enhance our understanding of Se metabolic pathways and ultimately support biofortification strategies of edible crops to address Se deficiency in humans.

**Table 1:** Summary of techniques and main findings in most studied Se hyperaccumulators species

	Approach	Species			
		*Astragalus bisulcatus*	*Stanleya pinnata*	*Cardamine violifolia*	*Neptunia amplexicaulis*
Se accumulation and distribution	ICP-AES	Up to 13,685 µg Se g^−1^ DW in leaves (Sura-de Jong et al. 2015)	> 4000 µg Se g^−1^ DW in young leaves (Galeas et al. 2006)	3700 µg Se g^−1^ DW in leaves (Both et al. 2018)	13,600 µg Se g^−1^ DW in young leaves (Harvey et al. 2020)
	SEM–EDS	Selenium concentrated in trichomes in young leaves (Freeman et al. 2006)	Selenium concentrated in vacuoles of epidermal cells (Freeman et al. 2010)	Unknown	Selenium concentrated in veins of young leaves (Harvey et al. 2020)
	Laboratory XFM	Unknown	Unknown	Unknown	Highest concentration in seed pods, young leaves and tap root (Harvey et al. 2020)
	Synchrotron XFM	Selenium concentrated in trichomes of leaf edges (Freeman et al. 2006)	High concentration in leaf edges (Freeman et al. 2006)	Selenium concentrated in root and shoot apical meristems (Both et al. 2020)	Unknown
Chemical speciation	XAS	MeSeCys in trichomes (Freeman et al. 2006)	MeSecys in globular structures in leaves (Freeman et al. 2006)	Mainly organic Se with a C-Se-C configuration in the shoot (Both et al. 2020)	MeSeCys and SeMet in similar proportions in young leaves (Harvey et al. 2020)
	LC–MS	MeSeCys and γ-glutamyl-MeSeCys in trichomes (Freeman et al. 2006)	MeSeCys (88%) and SeCT (12%) in leaf edges (Freeman et al. 2006)	Mainly selenolanthionine (Both et al. 2018)	MeSeCys and SeMet in the leaves (Harvey et al. 2020)
Molecular mechanisms	Transcriptomic analyses	High constitutive expression of *SULTR* transporters (Cabannes et al. 2011)	High constitutive expression of Se metabolism related enzymes (Freeman et al. 2010)	Up-regulation of ASKs and a PAPS family protein (Rao et al. 2020)	Unknown

## *Author contribution statement*

KPI wrote and edited the manuscript. MAH, HHH, MGMA CXC and PDE reviewed and edited the manuscript. AVDE reviewed, edited, and supervised the investigation.

## Data Availability

The datasets generated during and/or analysed during the current study are available from the corresponding author on reasonable request.

## Abbreviations

ATPS: Adenosine triphosphate sulfurylase
APSR: Adenosine 5′-phosphosulfate reductase
DMDSe: Dimethyldiselenide
DMSe: Dimethylselenide
MeSeCys: Methyl-SeCys
SeCys: Selenocysteine
SeMet: Selenomethionine
SMT: Selenocysteine methyltransferase
SULTR: Sulphur transporter
XANES: X-ray Absorption Near Edge Structure
XFM: X-ray fluorescence microscopy
XRF: X-ray fluorescence
